# P-2279. Breakthrough Invasive Fungal Infections in Patients with Acute Myeloid Leukemia on Anti-Fungal Prophylaxis (2022)

**DOI:** 10.1093/ofid/ofae631.2432

**Published:** 2025-01-29

**Authors:** Julia M Nelson, Ahmed Abdul Azim, Tanaya Bhowmick, Anjali Majumdar, Pinki Bhatt, Elizabeth Schmidt, Jaclyn Hayman, Navaneeth Narayanan, Sana M Mohayya, Moulika Baireddy, Bhargav Vemulapalli

**Affiliations:** Rutgers Robert Wood Johnson Medical School, New Brunswick, New Jersey; Rutgers Robert Wood Johnson Medical School, New Brunswick, New Jersey; Rutgers Robert Wood Johnson Medical School, New Brunswick, New Jersey; Rutgers-Robert Wood Johnson Medical School, New Brunswick, New Jersey; Rutgers - Robert Wood Johnson Medical School, New Brunswick, New Jersey; Rutgers Robert Wood John University Hospital, Morristown, New Jersey; Rutgers Robert Wood Johnson Medical School, New Brunswick, New Jersey; Rutgers University Ernest Mario School of Pharmacy & Robert Wood Johnson University Hospital, New Brunswick, NJ; Robert Wood Johnson Barnabas Health, New Brunswick, New Jersey; Rutgers, Robert Wood Johnson Medical School, Ashburn, Virginia; Robert Wood Johnson University Medical School, Skillman, New Jersey

## Abstract

**Background:**

Patients with hematologic malignancies, particularly Acute Myeloid Leukemia (AML), are at high risk for invasive fungal infection (IFI). Mold Active-Primary Antifungal Prophylaxis (MA-PAP) is recommended in these patients to prevent IFI, which carries high morbidity and mortality risk. Very few studies have compared rates of breakthrough IFIs (bIFIs) in AML patients treated with different MA-PAP regimens.

**Figure:**
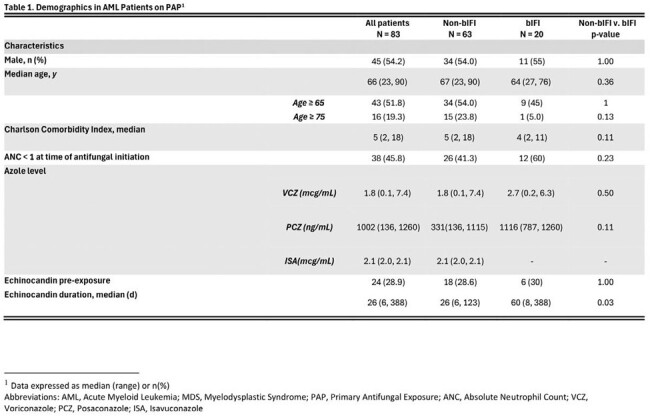

**Methods:**

This is a single-center, retrospective cohort study of adults with AML aimed at determining the efficacy of MA-PAP and the incidence and characteristics of bIFIs. We reviewed the medical records of patients ≥ 18 years old with a diagnosis of AML admitted to Robert Wood Johnson University Hospital (RWJUH) in 2022 and received MA-PAP with either Micafungin (MYC), Voriconazole (VCZ), Posaconazole (PCZ), or Isavuconazole (ISA) for > 7 days. bIFIs were classified as possible, probable or proven (based on the modified EORTC/MSG criteria) if they occurred after at least 7 days of MA-PAP initiation to within two weeks of discontinuation.

**Figure:**
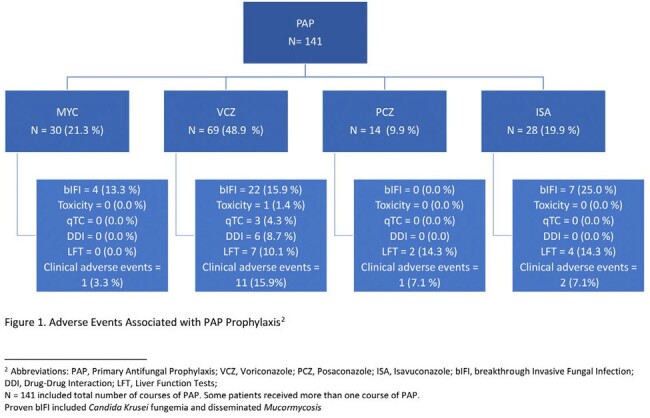

**Results:**

A total of 87 patients were included. Twenty patients (24.1%) experienced a total of 22 bIFI events, with 2 considered proven, 5 probable, and 15 possible. The incidence of bIFI was 15.4% for MYC, 18% for VCZ, 28% for ISA, and 0% for PCZ. 59.1% of bIFIs were in patients that were neutropenic for > 2 weeks at the time of bIFI. Proven bIFI were due to Candida Krusei and Mucormycosis. The mean length of hospital and ICU stay was the longest in the MYC group at 31 and 6 days respectively. Those who received VCZ experienced the highest number of reported adverse events (28, 41%) most often hepatotoxicity. Mortality was highest in PCZ (15.4%) followed by VCZ (11.5%).

**Figure:**
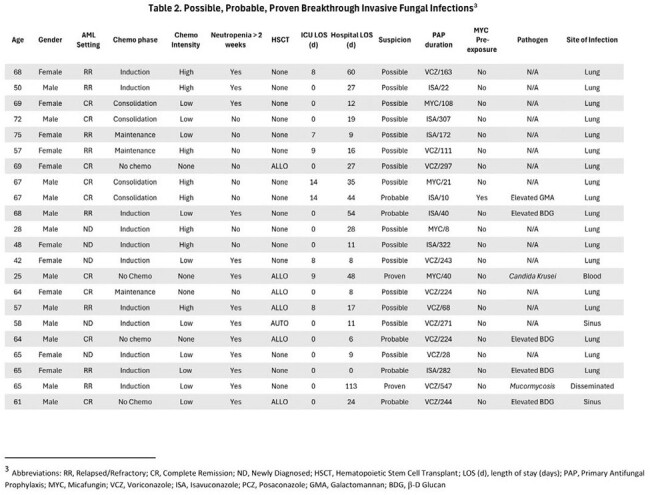

**Conclusion:**

Our institution experienced a high rate of bIFI, particularly among patients who received MA-PAP with VCZ or ISA. Prolonged neutropenia was an important risk factor for bIFI. Larger cohort studies are needed to accurately assess the risk factors and possibly mitigate the incidence of bIFI in this patient population on various MA-PAP. A comprehensive 10-year retrospective study is currently underway at RWJUH.

**Disclosures:**

Pinki Bhatt, MD, Sanofi: Grant/Research Support Navaneeth Narayanan, PharmD, MPH, BCIDP, Astellas: Honoraria|Beckman Coulter: Honoraria|Merck: Grant/Research Support|Shionogi: Grant/Research Support

